# Pandemic Avian Influenza and Intra/Interhaemagglutinin Subtype Electrostatic Variation among Viruses Isolated from Avian, Mammalian, and Human Hosts

**DOI:** 10.1155/2018/3870508

**Published:** 2018-05-17

**Authors:** Irene Righetto, Francesco Filippini

**Affiliations:** Molecular Biology and Bioinformatics (MOLBINFO) Laboratory, Department of Biology, University of Padova, Via Ugo Bassi 58/B, 35131 Padova, Italy

## Abstract

Host jump can result in deadly pandemic events when avian influenza A viruses broaden their host specificity and become able to infect mammals, including humans. Haemagglutinin—the major capsid protein in influenza A viruses—is subjected to high rate mutations, of which several occur at its “head”: the receptor-binding domain that mediates specific binding to host cell receptors. Such surface-changing mutations may lead to antigenically novel influenza A viruses hence in pandemics by host jump and in vaccine escape by antigenic drift. Changes in haemagglutinin surface electrostatics have been recently associated with antigenic drift and with clades evolution and spreading in H5N1 and H9N2 viruses. We performed a comparative analysis of haemagglutinin surface electrostatics to investigate clustering and eventual fingerprints among representative pandemic (H5 and H7) and nonpandemic (H4 and H6) avian influenza viral subtypes. We observed preferential sorting of viruses isolated from mammalian/human hosts among these electrostatic clusters of a subtype; however, sorting was not “100% specific” to the different clusters. Therefore, electrostatic fingerprints can help in understanding, but they cannot explain alone the host jumping mechanism.

## 1. Introduction

Influenza A viruses cause respiratory infections ranging from asymptomatic to deadly and can infect both birds and mammals, thus representing a dangerous threat to human health and poultry industry. Concerning humans, severe influenza A widespread outbreaks (pandemics) can result in the death of tens of million people worldwide, such as what occurred in 1918, 1957, and 1968 [1]. Wild ducks are the largest reservoir of avian influenza (AI) viruses that can also sporadically infect domestic birds and mammalian species including swine, cats, dogs, horses, and unfortunately also human hosts [2]. Influenza A viruses are classified based on subtypes of the two major capsid proteins and surface antigens haemagglutinin (HA) and neuraminidase. Functionally, haemagglutinin acts as a key contributor to changes in host specificity in AI viral infection [3]. Structurally, HA mature monomers consist of chains HA1 and HA2 produced by proteolytic cleavage of the unfolded precursor [4]. Mature monomers fold as trimers exposed at the viral surface and show a globular “head” (part of chain HA1) that includes a receptor-binding domain (RBD) and a vestigial esterase domain (VED); the RBD mediates docking to the host cell by binding sialic acids (SA) as cell entry receptors [4].

So far up to 18 major subtypes (H1 to H18) have been classified and deposited in public databases such as the Influenza Research Database (IRD) [5]; among all such subtypes, H5, H7, and H9 are of special interest to surveillance and characterization as top pandemic agents [6, 7]. In particular, H5N1 AI viruses show the broader host range and geographical spreading [8] and a well-known epidemiologic story with humans [9], while recently reassorted H7N9 and H9N2 also increased concern for jumping the host-species barrier, hence resulting in pandemic risk [10, 11]. A recent project developing an evidence-based risk assessment framework for influenza viruses in animals reviewed human cases naturally infected with AI viruses highlighting the fact that they especially spanned HA subtypes H5, H7, and H9 and to a minor extent H6 and H10 [12]. The transition from low pathogenic AI (LPAI) to high pathogenic AI (HPAI) has been reported for both H5 and H7 subtypes after their introduction into poultry, likely because of infection from wild birds in a mixed environment [13]. Current AI vaccines are mainly based upon eliciting the anti-haemagglutinin antibody response and thus antigenic drift and vaccine escape may depend on mutations in HA surface regions [4]. Surface variation has been related as well to modulation/change in host specificity, depending on increased binding to *α*2-6 SA, and thus improved affinity to the human host [14–16]. Recent crystallographic studies have provided molecular insights into some interactions between haemagglutinin and host receptors likely having enabled several AI virus subtypes to jump from avian to human hosts [17]. Indeed, surface epitope interactions can be modulated in several (and often contemporary) ways, depending on the variation of the multiple features (steric hindrance, electrostatic charge, polar or hydrophobic nature, etc.) that each residue in the surface region is endowed with. Interactions are modulated as well by the overall aggregate or synergistic effect resulting from changes in neighbouring residues.

We recently reported that when comparing the HA receptor-binding domain (RBD), electrostatic closeness can group haemagglutinins from different AI virus phylogenetic groups. In particular, H5 (which belongs to HA Group 1) was found to be quite closer to H9 (HA Group 2) than to H2 (same group) [18]. Deeper analysis performed on H5N1 clades and subclades unveiled electrostatic fingerprints that relate to clades evolution and spreading, and surface charge redistribution was suggested to be likely involved in antigenic drift events [18]. More recently, we showed that relationship between electrostatic fingerprints and virus evolution also concerns H9N2 clades, confirming that this is a general hallmark for AI viruses, rather than a special feature of H5N1 [19]. Such findings on “pandemic risk” AI viruses prompted us to further investigate on electrostatic distance and grouping among model AI subtypes, and in particular on possible differences in electrostatic clustering and sharing of fingerprints among viruses isolated from avian and human/mammalian host.

Indeed, electrostatics is an important player in the modulation of interactions at all subcellular levels. In particular, electrostatic interactions are ubiquitous in proteins and dictate stability and function. As reported by Ritchie and Webb [20] structural and electrostatic factors are crucial in the affinity and specificity of macromolecular interactions, protein folding, and chemical reactivity. For example, electrostatics may regulate the interaction of proteoliposomes with lipid membranes [21], and specific electrostatic interactions between charged amino acid residues regulate binding of (i) von Willebrand factor to blood platelets [22] and (ii) of GTP-binding proteins of the RAS superfamily with downstream effector proteins [20]. It is well-known that clusters of charged and polar residues that are located at protein-protein interfaces may enhance complex stability [23]. Therefore, alterations in protein electrostatics also play an important role in pathogenesis, for instance, by regulating self-aggregation mechanisms underlying a number of neurodegenerative disorders [24, 25].

Electrostatics is able to keep influenza virus Matrix Protein M1 conformation stable at different pH values [26], and diverse electrostatic characteristics at host-interaction interfaces are involved in different modes of virus pathogenesis [27]. This evidence suggests that interfering with electrostatics can help developing antiviral drugs; for instance, P20A drug inhibits HIV-1 fusion through its electrostatic interaction with the distal region of the gp41 fusion core [28]. Electrostatics is also studied to develop inhibitors for the influenza virus neuraminidase [29]. Methods for driving alteration of the electrostatic properties are considered for designing proteins with optimized binding and activity [30]; for instance, electrostatic optimization was used for modulating protein-protein association rates [31].

However, and in spite of being so important for protein function and dysfunction, electrostatics is only one player among other (equally important) modulators of the protein surface features (hence of protein interactions). Indeed, solvent accessible surface area and the balance between hydrophilic and hydrophobic patches and side chain orientation at specific linear or conformational epitopes may play a pivotal role as well. Therefore, having found and confirmed relationship with virus clades evolution [18, 19], we wondered to know whether electrostatics might be (i) the major player also in the modulation of host specificity, (ii) an important (but not the major) player, contributing with others to regulate host specificity, or (iii) a poorly relevant feature in this context.

In this work, we studied electrostatic variation among four HA subtypes, of which two (H5 and H7) representing a model for “pandemic” subtypes and two (H4 and H6) for “non/poorly pandemic” ones. We started by comparative analysis of HA1 chains, as HA1 contains the RBD, where electrostatic fingerprints were found [18, 19], which is endowed with SA receptors [4]. Then, the analysis was performed by progressively zooming in the HA1 and RBD subregions, and relevant antigenic sites.

## 2. Materials and Methods

### 2.1. Sequence Retrieval and Structural Modeling

Data were obtained from the NIAID Influenza Research Database (IRD) [5] through the website at https://www.fludb.org. Target protein sequences were modeled on best available structure templates using SWISS-MODEL [32]. Model quality was checked via QMEAN server [33].

### 2.2. Electrostatic Analyses

Isopotential contours were calculated using UCSF Chimera v. 1.11.2 [34] via Adaptive Poisson-Boltzmann Solver (APBS) [35] through Opal web server. Isopotential contours were then plotted at ±2*k*_*B*_*T*/*e*. PDB2PQR [36] was used to assign partial charges and van der Waals radii according to the PARSE force field [37]. Interior *ε*_*p*_ = 2 and *ε*_*s*_ = 78.5 were chosen for, respectively, the protein and the solvent [30, 38, 39], *T* = 298.15 K. Probe radius for dielectric surface and ion accessibility surface were set as *r* = 1.4 Å and *r* = 2 Å, respectively. Electrostatic distance (ED):

(1) $$\begin{array}{c} \text{Electrostatic distance }D_{a,b}=\sqrt{{2-2SI}_{a,b}} \end{array}$$

was calculated using the Hodgkin index at the WebPIPSA server [40], which can accept (November 2017 version) up to seventy protein structures. Rigid body superposition was performed and the electrostatic map was plotted onto the molecular surface using UCSF Chimera v. 1.11.2.

## 3. Results and Discussion

### 3.1. Analysis of Electrostatic Distance in H5 Subtype Viruses Isolated from Avian, Mammalian, and Human Hosts

In two recent studies, electrostatic distance (ED) analysis of the different AI virus clades and subclades was used to integrate phylogenetic analyses, and comparative analysis of electrostatic isocontours unveiled “charge redistribution” events at the RBD surface that suggested a molecular rationale for evolutionary drift underlying adaptation and spreading of successful clades in H5N1 and H9N2 [18, 19]. This prompted us to check whether—in addition to being involved in antigenic drift—electrostatic variation might play a role in avian to mammalian/human host jump. Indeed, finding any kind of association between host jump and specific electrostatic group(s) might be of help to surveillance programs in that providing fingerprint(s) predictive for pandemic potential. We started our analysis by H5 subtype as the AI virus subtype model for well-known pandemic track and because of the aforementioned pilot work on H5N1 electrostatics [18]. The NIAID Influenza Research Database (IRD) [5] contains several hundreds of haemagglutinin sequences belonging to H5N1 viruses isolated from avian hosts (a-host) and a few tens from human/mammalian host (h/m-host). Once electrostatic fingerprints are demonstrated to tag clades evolution [18, 19], we aimed at investigating sorting of h/m-host viruses.

**Figure 1:**
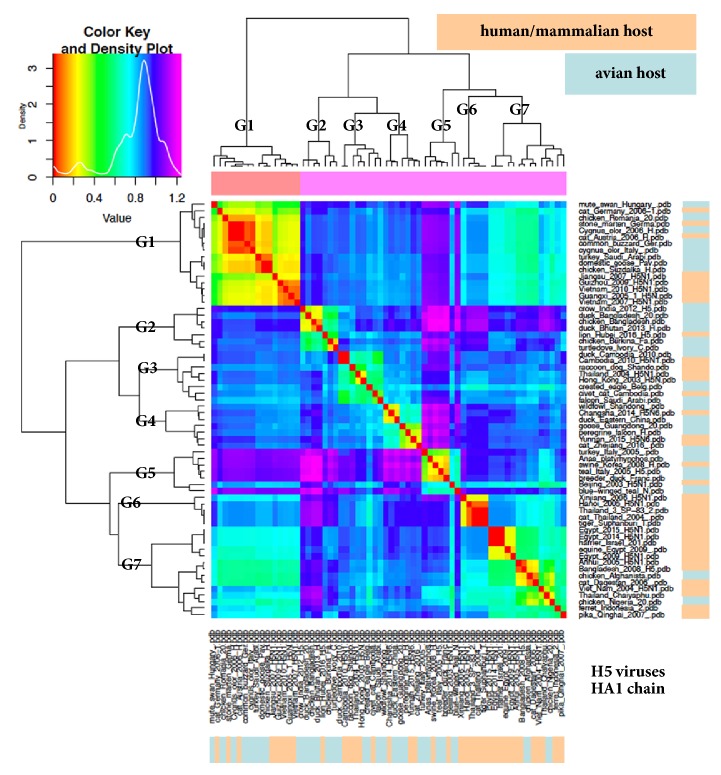
ED analysis (heatmap) of a sixty-four haemagglutinin HA1 chains dataset from H5 viruses. As reported in the density plot, the warmer the colour, the lower the ED. Group numbers are established as explained in the main text; right and bottom bars highlight distribution of viruses isolated from either avian or human/mammalian host, according to indicated colour coding.

We populated a sixty-four dataset for ED analysis (see Materials and Methods for number) by overrepresenting (thirty-five) h/m-host subpopulation with respect to a-host viruses (twenty-nine). In early analyses, the HA1 mature chain for each haemagglutinin sequence was considered. Since the input for the ED analysis consists of pdb structures rather than sequences, a structural model for each HA1 chain was obtained by homology modeling with high confidence, because of the very high identity with the available structural template. This notwithstanding, model quality was assessed via QMEAN [18] prior to ED analyses.  Figure 1 depicts as a “heatmap” the ED analysis for the sixty-four H5N1 HA1 chains, which seemingly are sorted into seven electrostatic clusters. In order to properly interpret heatmap figures, it should be mentioned that lower to higher ED transition is colour coded in the “density plot.” Furthermore, a doubled “epogram” (i.e., an electrostatic potential cladogram), at both the left and upper sides of the heatmap, highlights ED-based clustering. In Figure 1, electrostatic clusters can be recognized and hereafter numbered as “groups” corresponding to square areas starting by North-West position (Group 1) and proceeding along the Southeast direction. Warm colours highlight the low ED among the sixteen viruses of an electrostatically very homogeneous Group 1, equally populated by a-host and h/m-host viruses, of which five are from humans. Group 2 includes seven viruses, of which only one is from mammalian (lion) host. In Group 3 (eight viruses), Group 4, and Group 5 (seven viruses each) two to four h/m-host viruses per group are present. In the last two groups, h/m-host viruses represent the major subpopulation: five out of five in Group 6 and ten out of thirteen in Group 7, including several viruses isolated from humans. In order to confirm the homogeneity in the grouping as defined by ED analysis, a deeper analysis of electrostatic isocontours for all sixty-four structures was performed.

### 3.2. Specific and Homogeneous Electrostatic Isocontours in the H5 Dataset Are Associated with Electrogroups Identified via ED Analysis

**Figure 2:**
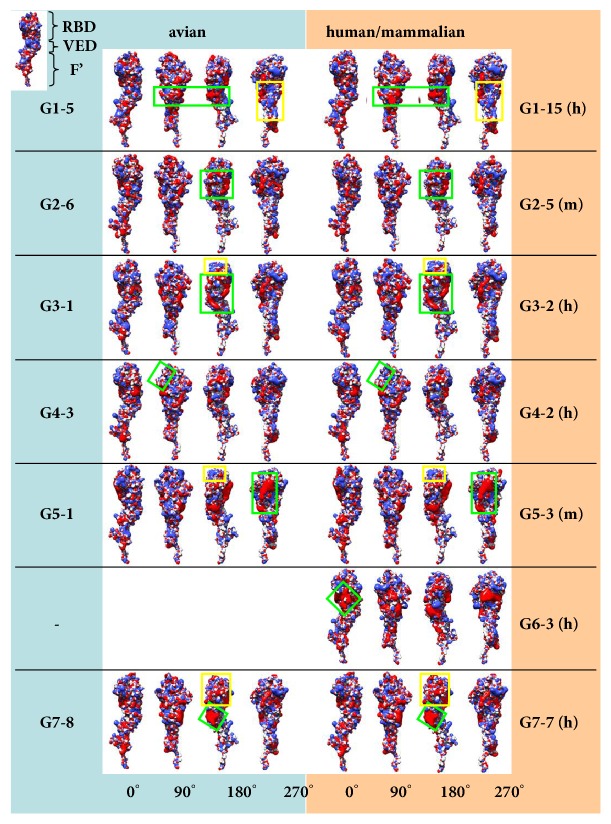
Electrostatic isocontours and fingerprints for haemagglutinin HA1 chains from H5 virus couples, representative for each group (as defined by ED analysis presented in Figure 1). Avian host-virus is missing for G6. Four, 90° stepwise rotation views are provided for each isocontour. Colour coding associated with avian (a), human (h), or other mammalian (m) hosts is the same as in heatmap figures. The three main subregions of the HA1 chain are depicted at the top-left corner. For specific virus numbering please refer to the complete dataset as presented in Supplementary Figure S1. Fingerprints are highlighted by green and yellow boxes. Group 1 (G1) exhibits a negative charge (red) at the RBD-VED intermediate region (green box). In addition, this region shows a positive charge (blue), extending towards the F' region (yellow box). Fingerprint in G2 is shaped as a negatively charged circle with inner central spot at the RBD (green box). A similar signature is found in G3, where the negative circle extends towards the F' region (green box). G3 viruses also show a positive charge at the top of RBD (yellow box, shared with G5), whereas this region is less positive or even contains negative charge in other groups. G5 also shows a peculiar, very negative region at the RBD (green box). All groups but G4 share two or three positive spots at the top-left side (90° view) of the RBD; this region is green boxed in G4 to highlight missing dots. G6 viruses show a negative region located at the RBD-VED interface with rhomboid shape and a central, neutral (white) spot peculiar to this group (green box). A large negative area at the RBD + VED is evident in G7, being homogeneously red at the VED (green box) and dispersed at the RBD (yellow box).

The electrostatic isocontour for each HA1 structural model was obtained (see Materials and Methods), and four 90°-stepwise views are presented as a full-view flat picture for each isocontour. A multipage figure, depicting the complete analysis with all sixty-four viruses, is presented as Supplementary Figure S1, while “seed” (Figure 2) only includes a representative a-host-h/m-host couple for each group. A preliminary visual inspection of the complete groups in Figure S1 suggests soon that clustering by ED analysis is confirmed by evident intragroup homogeneity and intergroup difference of electrostatic isocontours. However, this is further confirmed by group-specific fingerprints, described in detail in the caption to Figure 2, and boxed for all sixty-four viruses also in Supplementary Figure S1.

### 3.3. Analysis of Electrostatic Distance in H7, H4, and H6 Subtype Viruses Confirms Sorting of Viruses Isolated from Humans or Other Mammals in Multiple Electrogroups

The ED analysis performed with H5 was repeated with a same-size dataset of viruses from another AI subtype having a well-known track of infections in humans and other mammals, that is, H7 (see introduction). Several different electrostatic groups are found also with H7; in particular, the three largest groups (G1–G3) reveal a relatively low intragroup ED (warm colours, Supplementary Figure S2). Retrieved H7 viruses from h/m-host in this dataset are twenty-four, of which twenty-one having infected humans. Spreading of such strains over electrostatic groups resembles evidence observed with H5: all groups contain viruses from h/m-hosts, but in some groups they are poorly represented (two in G1, three in G2 and one in G4) and in others they show high numbers (twelve in G3) or even miss a-host counterparts (G5). For comparison to AI subtypes with a poor story of infections in mammals, ED analysis was performed with two further HA1 datasets from H4 and H6 subtypes (Supplementary Figure S2). All viruses from mammalian host available at IRD for H4 (three, isolated from swine) and for H6 subtype (two, one from canine and one from swine) were used. Both heatmaps show six different electrostatic groups, a number in the average with H5 (seven) and H7 (five). In H4, the three viruses from swine are sorted to three different electrogroups (G1, G3, and G6); the same happens with H6, where the virus from canine belongs to G4 and the one from swine to G6.

### 3.4. “Zoom In” Analysis of ED in Haemagglutinin Subregions

**Figure 3:**
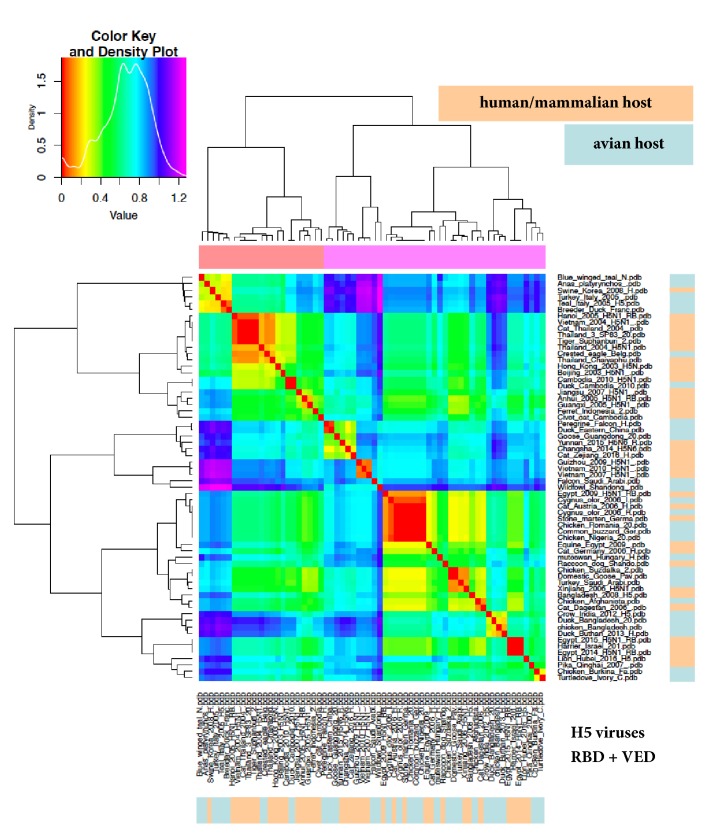
ED analysis (heatmap) of the RBD + VED subregion of haemagglutinin. H5 dataset and colour coding as in Figure 1.

**Figure 4:**
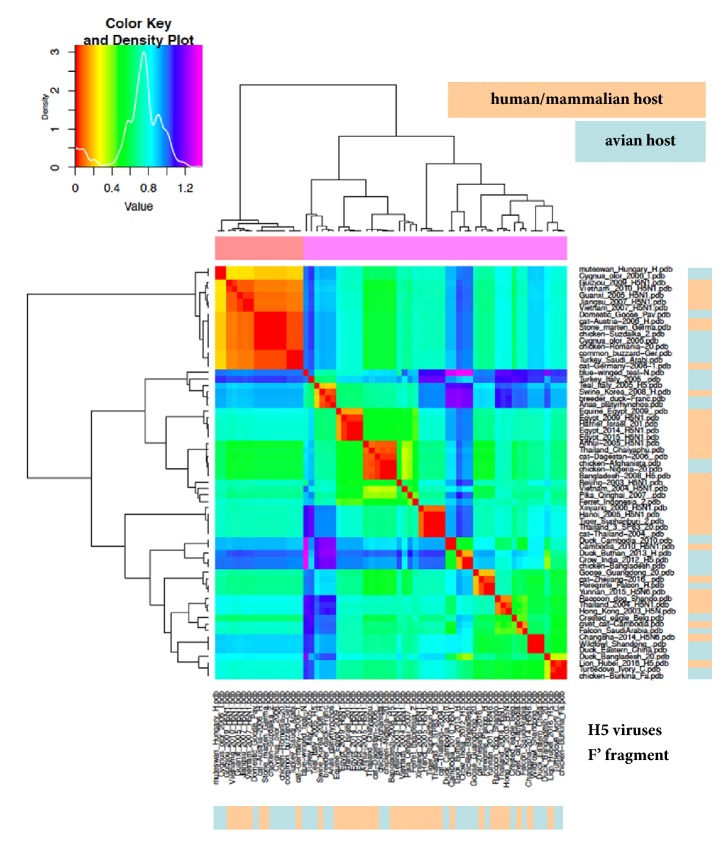
ED analysis (heatmap) of the F' fragment of haemagglutinin. H5 dataset and colour coding as in Figure 1.

Considering that, at least so far, electrostatic fingerprints for clades evolution were found to be restricted to the RBD [18, 29], we repeated the ED analyses with the RBD + VED part of HA1 (i.e., cutting the F' fragment contribution off).  Figure 3 depicts the results for H5 viruses: once again, several groups are found in which a-host and h/m-host viruses are mixed. Grouping comparison for RBD + VED versus HA1 shows only partial agreement, as some rearrangement is found. This is not surprising at all, because of the different electrostatic features in haemagglutinin subregions [18]. Indeed, the HA1 chain consists of RBD + VED and the F' fragment and thus we also performed the F' fragment ED analysis, finding a group clustering quite different with respect to RBD + VED (see Figure 4). When iterating the RBD + VED ED analysis with H7, H4, and H6 viruses (see Supplementary Figure S3), partial agreement with HA1 analysis was confirmed, as well as no separation among a-host and h/m-host viruses.

**Figure 5:**
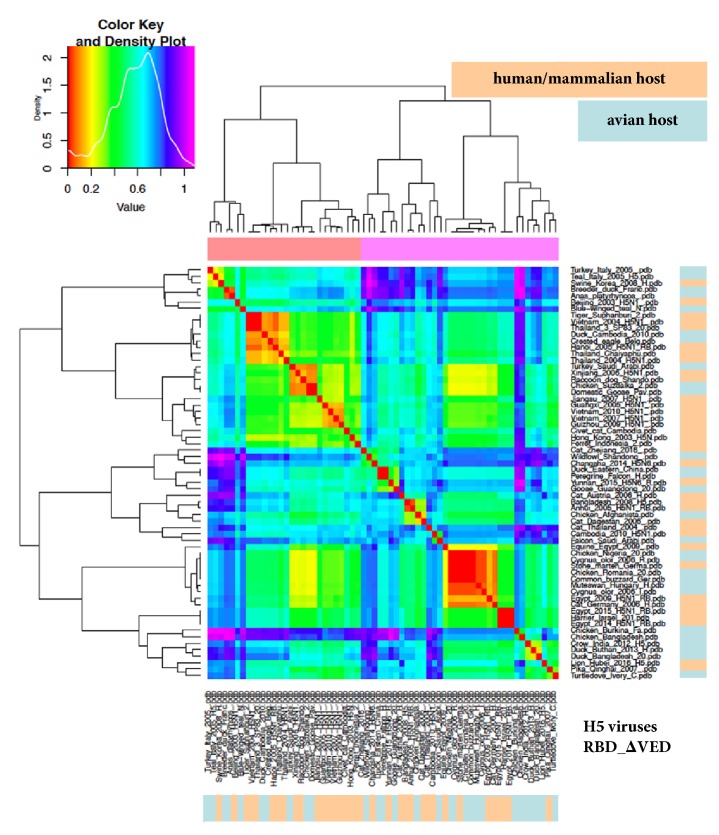
ED analysis (heatmap) of the RBD_ΔVED subregion of haemagglutinin. H5 dataset and colour coding as in Figure 1.

**Figure 6:**
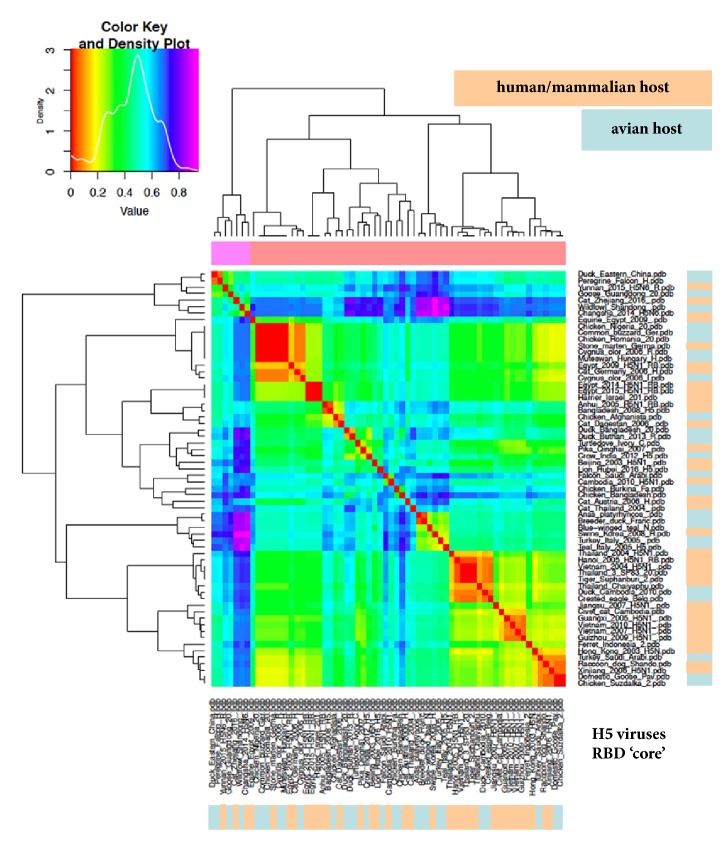
ED analysis (heatmap) of the strictly antigenic, “core” RBD subregion of haemagglutinin (encompassing 130-loop to 220-loop). H5 dataset and colour coding as in Figure 1.

Given that the most important antigenic regions involved in binding to SA and thus in host specificity and jump events are restricted to the* sensu stricto* RBD subregion, we aimed at further “zooming in” the analysis around such epitopes. Therefore, the analysis was repeated by cutting also the VED region off. Clustering in H5 for the RBD alone is depicted in Figure 5 and, once again, groups consisted of mixed a-host and h/m-host viruses. Finally, we focused ED analysis on the “core”, antigenic region of the RBD, encompassing subregion from 130- and 150-loop to 190-helix and 220-loop: Figure 6 shows that groups still consist of mixed a-host and h/m-host viruses. When analysing such RBD “core” subregion also in terms of hydropathy (as reported [18]), a-host and h/m-host viruses showed quite similar profiles (not shown).

## 4. Conclusions

When using “avian influenza virus” keywords for a PubMed search (March 2018), more than 43000 published papers can be retrieved. Papers are still thousands with additional search keywords such as “pandemic” or “evolution” or hundreds when adding, for example, “antigenic drift”, “immune escape”, or “host specificity”. The most of such works were based on wet biology analyses and/or sequence based phylogenetic trees; however, in spite of intriguing evidence having emerged from multiple, very good works, no paper could place any final explanation, or even to clearly identify the major player for viral clade evolution and spreading or for host specificity shift. Considering that structure based, more systematic analysis was needed, we recently focused on comparative electrostatic analysis of the haemagglutinin surface. This allowed us to demonstrate that, in H5N1, electrostatic fingerprints and a progressive change in charge distribution are clearly related to clades and subclades evolution and spreading in different AI types [18]. Then, validation in H9N2 clarified that such relationship represents a general mechanism in AI virus evolution, with RBD electrostatics playing an important role in antigenic drift/immune escape, as well as in the evolutionary success of circulating clades [19]. In this work, we performed instead the first systematic analysis of the surface electrostatics versus host specificity relationship, by contemporary comparison of datasets from four AI virus subtypes and zoom in dissection of haemagglutinin. When performing ED analysis with different haemagglutinin subregions, viruses isolated from avian or human/mammalian host were found to be sorted into multiple electrostatic groups, including mixed a-host and h/m-host viruses. This happens with both AI subtypes having “pandemic story” (H5 and H7) or a poor story of infections in mammals (H4 and H6). Most importantly, even when progressively zooming in relevant antigenic epitopes of the RBD, this does not support any “unique” or major role for electrostatics in determining/altering host specificity. However, when considering relative distribution among the electrostatic groups, some “preference” is evident with both H5 and H7 (where numbers of retrieved viruses from h/m-hosts allow for relative distribution comparison). In both subtypes, one electrostatic group is fully populated by viruses from h/m-host; others contain several such viruses and finally, in some groups, viruses from an avian host are the major population. Preferential grouping was confirmed at all levels, that is, in next “zoom in” ED analyses with progressively smaller fragments focusing around the most antigenic 130-loop to 220-loop fragment. Therefore, although no “predictive” (for host jump) electrostatic isocontour is found, preference for some clusters over others suggests electrostatic changes to be involved—together with other surface features—in the modulation of host specificity. Among further features known to modulate surface interactions, hydropathy did not show any profile specifically associated with either avian or mammalian host. A very recent work [41] highlighted the relevance for host specificity of some changes at the 130-loop, even if it could not find out a “final explanation” and a unique rationale for this mechanism. Indeed, Timofeeva and coworkers reported [42] that the decrease of the positive electrostatic charge in the vicinity of RBD epitopes involved in immune escape could also lead to a lowering of the affinity to SA analogs of cell receptors. Aforementioned works neither excluded nor could demonstrate electrostatics to play a major role in the modulation of host specificity, as systematic analyses are needed to provide general conclusions. Therefore, proteome-wide evidence from this work could bring some clarity on host specificity, confirming that electrostatics is likely a relevant coplayer, while suggesting that it is not the major one. In other words, electrostatic changes are seemingly unable “to drive” host specificity and they are likely able—together with other surface features—“to modulate” specificity. Among further surface features, hydropathy was found to be less important in the context, because of no relationship to any sorting preference. In conclusion, next investigations on changes in surface electrostatics should be studied in combination to local changes in, for example, solvent accessible surface area and/or specific linear and conformational motifs. Furthermore, this could be integrated by docking simulations, for predicting changes in relative affinities to *α*-2, *α*-3, *α*-2, and *α*-6 SA, possibly underlying host jump and pandemic events.

## Supplementary Materials

Supplementary Materials Figure S1: electrostatic isocontours and fingerprints for haemagglutinin HA1 chains from the complete dataset of sixty-four H5 viruses (see ED analysis presented in Figure 1). Four, 90° stepwise rotation views are provided for each isocontour. Viruses were isolated from (a) avian; (h) human; (m) other mammalian hosts. Virus numbering is based on the group (G1 to G7) and on its position in the heatmap. Fingerprints are highlighted by green and yellow boxes. Figure S2: ED analysis (heatmap) of a sixty-four haemagglutinin HA1 chains dataset from H7, H4, and H6 viruses. Group numbering and colour coding as in Figure 1. Figure S3: ED analysis (heatmap) of a sixty-four haemagglutinin RBD + VED subregions from H7, H4, and H6 viruses. Group numbering and colour coding as in Figure 3.
